# Clostridium lapidicellarium sp. nov. and Clostridium moutaii sp. nov., two species isolated from fermentation cellar-producing sauce-flavour Chinese baijiu

**DOI:** 10.1099/ijsem.0.006580

**Published:** 2024-11-19

**Authors:** Fan Yang, Hui Wang, Liang-Qiang Chen, Nan Zhou, Jian-Jun Lu, Xiu-Xin Pu, Bo Wan, Li Wang, Shuang-Jiang Liu

**Affiliations:** 1 Kweichow Moutai Distillery Co., Ltd., Zunyi 564501, PR China; 2 State Key Laboratory of Microbial Resources and Environmental Microbiology Research Center, Institute of Microbiology, Chinese Academy of Sciences, Beijing 100101, PR China; 3 State Key Laboratory of Microbial Technology, Shandong University, Qingdao 266237, PR China

**Keywords:** *Clostridium lapidicellarium*, *Clostridium moutaii*, *Clostridium sensu stricto* (cluster I), pit mud, sauce-flavour Chinese *baijiu*

## Abstract

*Clostridium* is an important microbial component in pit mud due to its ability to produce alcohol and short-chain fatty acids. This study presents the characterization and taxonomy of two Gram-stain-positive, strictly anaerobic, rod-shaped mesophilic bacterial strains, designated MT-113^T^ and MT-5^T^, isolated from pit mud in a fermentation cellar used for producing sauce-flavour Chinese baijiu. Phylogenetic analysis based on genome and 16S rRNA gene sequences of strains MT-113^T^ and MT-5^T^ indicates their affiliation with the genus *Clostridium sensu stricto* (Cluster I of the Clostridia), with *C. luticellarii* FW431^T^ and * C. aromativorans* WLY-B-L2^T^ as the closest related species. The major cellular fatty acids (>10.0%) of both strains are C_14 : 0_ and summed feature 1 (iso-C_15 : 1_ h and/or C_13 : 0_ 3-OH). The G+C molar contents of the complete genomes for strains MT-113^T^ and MT-5^T^ are 35.84 and 32.74 mol%, respectively. The average nucleotide identity and average amino acid identity values between strains MT-113^T^, MT-5^T^, *C. aromativorans* WLY-B-L2^T^ and *C. luticellarii* FW431^T^ range from 79 to 85%. The primary products of glucose fermentation by MT-113^T^ are acetic, butyric and isovaleric acids, while those of MT-5^T^ are acetic, isobutyric, butyric and isovaleric acids. Based on their phenotypic, chemotaxonomic and phylogenetic characteristics, strains MT-113^T^ (=CGMCC 1.18018^T^ = JCM 36532^T^) and MT-5^T^ (=CGMCC 1.18016^T^ = JCM 36530^T^) are proposed as the type strains of two novel species of the genus *Clostridium*, namely *Clostridium lapidicellarium* sp. nov. and *Clostridium moutaii* sp. nov., respectively.

## Introduction

The genus *Clostridium*, a representative of the family *Clostridiaceae*, was initially proposed by Prazmowski in 1880, with *Clostridium butyricum* as the type species [1]. Approximately 250 *Clostridium* species with valid names are currently documented in the List of Prokaryotic Names with Standing in Nomenclature (http://www.bacterio.net). The genus is characterized by anaerobic respiration, spore formation, Gram-positive staining, low G+C content and the production of short-chain organic acids during fermentation [2,3]. Nearly half of the species with valid names belong to the *C. butyricum* lineage, recognized as *Clostridium sensu stricto* (Cluster I) [4,5]. Notably, species previously assigned to other genera such as *Eubacterium*, *Anaerobacter* and *Sarcina* were once classified within *Clostridium* Cluster I [1,4, 5]. *C. butyricum*, the type species, is extensively studied for its ability to produce significant quantities of short-chain organic acids [6,7]. In addition to *C. butyricum*, strains such as *C. aromativorans*, *C. luticellarii*, *C. celerecrescens*, *C. cochlearium*, *C. carboxidivorans*, *C. sporogenes*, *C. sartagoforme*, *C. thermopalmarium* and * C. aurantibutyricum* have been isolated from pit mud used in producing baijiu, a traditional Chinese fermented alcoholic beverage [8,11]. Sauce-flavour Chinese baijiu production involves natural fermentation in stone cellars with a 20–30 cm layer of mud at the base. During the year-long spontaneous solid-state fermentation process, the cellars maintain anaerobic conditions and high ethanol concentrations, fostering diverse populations of obligate and facultative anaerobic microbes [12]. *Clostridium* is a significant microbial component in pit mud due to its ability to generate short-chain fatty acids, ethanol and other carbohydrate derivatives, influencing the quality and aroma profiles of the liquor [9,10, 13,17]. This study details the isolation and characterization of two strictly anaerobic, Gram-positive bacterial strains, MT-113^T^ and MT-5^T^, from pit mud in fermentation cellars continuously used for producing sauce-flavour Chinese baijiu.

## Methods

### Sample collection and treatment

Strains MT-113^T^ and MT-5^T^ were isolated from pit mud collected from the bottom layer of Chinese baijiu fermentation cellars at Kweichow Moutai Co., Ltd., located in Renhuai City, Guizhou Province, PRC (27.51 N, 106.22E). The pit mud samples were promptly transferred to an anaerobic workstation (Electrotek AW400SG, West Yorkshire, UK) with a gas mixture of CO2/H2/N2 (5%/10%/85%). Subsequently, 5 g of sludge was suspended in 50 ml of 0.01 M PBS (catalogue number P1022, Solarbio), homogenized and filtered through a 40 µm cell sieve (Falcon). The filtrate was serially diluted by factors of 10 from 10^−1^ to 10^−7^ and dilutions from 10^−4^ to 10^−7^ were plated on modified Clostridial Growth Medium (mCGM) [18] solidified with 1.5% (w/v) agar, followed by anaerobic incubation at 37 °C for 3–7 days.

### Culture media and preservation

Initially, the strains were cultured using mCGM medium; however, subsequent investigations revealed that they could also grow in Reinforced Clostridium Medium (RCM) [10], albeit at a slower rate compared to mCGM. The composition of mCGM per litre includes 20 g glucose, 10 g tryptone, 10 g yeast extract, 3.66 g sodium butyrate, 4.55 g sodium acetate trihydrate, 2 g (NH_4_)_2_SO_4_, 1 g KH_2_PO_4_, 0.5 g K_2_HPO_4_, 0.1 g MgSO_4_·7H_2_O, 0.015 g FeSO_4_·7H_2_O, 0.01 g MnSO_4_·H_2_O, 0.01 g CaCl_2_, 0.002 g CoCl_2_ and 0.002 g ZnSO_4_. The medium was prepared with deoxygenated distilled water, adjusted to pH 7.0 and autoclaved at 115 °C for 30 min. Bacterial purity was confirmed through microscopic observation of morphological uniformity and 16S rRNA gene sequencing analysis. Before long-term storage at −80 °C in 20% (v/v) glycerol, the strains were further purified by subculturing twice on a fresh medium. All type strains designated in this study are catalogued at the China General Microbiological Culture Collection Center (CGMCC, China) and the Japan Collection of Microorganisms (JCM, Japan), with accession numbers provided in the species description section.

### Cell physiological and biochemical taxonomic determinations

Cell morphology was investigated using transmission electron microscopy (TEM, JEM-1400; JEOL) following anaerobic cultivation on mCGM agar plates at 37 °C for 3 days. Gram staining adhered to the manufacturer’s instructions using a Gram-staining kit (catalogue number G1060, Solarbio). The temperature range for growth was evaluated at 4, 15, 20, 30, 37, 40, 45, 60 and 70 °C, after a 3 day incubation in mCGM. Similarly, the pH range for growth was assessed at pH values of 4.0, 5.0, 5.5, 6.0, 6.5, 7.0, 7.5, 8.0, 8.5, 9.0, 10.0 and 11.0 by adjusting the mCGM pH with specific buffers prior to sterilization [19]. NaCl tolerance was tested in mCGM medium supplemented with NaCl from 0 to 5% (w/v) at 0.5% intervals. Cell growth was determined by measuring turbidity at 600 nm (OD600) using a UV/visible spectrophotometer (SPECORD205; Analytik Jena). Aerobic growth was assessed by incubating mCGM agar plates at 37 °C for 3 days. Although the isolated strains are associated with baijiu brewing, further research is needed to optimize methods to enhance or control their growth, which is crucial for advancing this field. Antibiotic susceptibility testing was performed using the single-disc diffusion method with commercial antibiotic discs [20]. The diameter of the inhibition zones was measured using 8 mm test discs (Bioroyee), containing the following antibiotics (µg per disc unless otherwise stated): chloramphenicol (30), rifampin (5), polymyxin B (300 IU), streptomycin (10), tetracycline (30), spectinomycin (100), carbenicillin (100), vancomycin (30), erythromycin (15), ciprofloxacin (5), penicillin (10), azithromycin (15), clindamycin (2), amoxicillin (10), neomycin (30), kanamycin (30), gentamicin (10), cefixime (5), cephalexin (30) and ampicillin (10). The inhibitory zones were evaluated following anaerobic culture at 37 °C for 3 days, with all experiments conducted in triplicates.

Carbon source utilization was evaluated using a 96-well Biolog AN microplate (Biolog Inc., USA), which contains 95 different carbon substrates [21]. Physiological and biochemical characteristics were determined using the Rapid ID 32A Anaerobe Identification Kit and the API ZYM Kit (both from BioMérieux, France) in accordance with the manufacturer’s instructions. Bacterial strains were cultured in liquid mCGM medium for 3 days, after which cells were collected for subsequent analysis.Cellular fatty acid methyl esters were prepared and analyzed using an HP 6890A series GC system (Agilent Technologies), following the standard protocol for the Sherlock Microbial Identification System (MIDI system, version 6.0) [22]. Polar lipids were fractionated using two-dimensional TLC on silica gel-coated TLC plates (10×10 cm; Merck). Chromatography employed chloroform/methanol/water (65 : 25 : 4, v/v) for the first dimension and chloroform/methanol/acetic acid/water (80 : 12 : 15 : 4, v/v) for the second dimension [23]. Total lipids were visualized using a 10% ethanolic molybdophosphoric acid solution (Sigma). Aminolipids were detected with a 0.4% ninhydrin solution (Sigma) in butanol, phospholipids (PLs) with the Zinzadze reagent (1.3% molybdenum blue spray reagent; Sigma) and glycolipids (GLs) with a 0.5% *α*-naphthol reagent.

Bacterial strains were cultured in RCM broth for 3 days at 37 °C under strictly anaerobic conditions. A 1 ml culture sample was then extracted with 1 ml of ethyl acetate. The resulting supernatant was analysed using GC-MS (GC-MS-QP2010 Ultra, Shimadzu) to assess the organic acids and metabolites produced by the strains. The GC-MS system featured an autosampler and a DB-wax capillary column (30 m in length, 0.25 mm inner diameter, 0.25 µm film thickness; Shimadzu). The oven temperature was programmed to increase from 80 to 140 °C at a rate of 20 °C min^−1^, with a 1 min hold; subsequently, it was raised to 290 °C at 3.5 °C min^−1^ with a 15 min hold. Sample injection (1 µl) was conducted at 280 °C, with helium gas as the carrier at a flow rate of 1.2 ml min^−1^. The electron impact was set at 70 eV [24].

### Phylogenetic analysis

Phylogenetic analyses were conducted using 16S rRNA gene sequences obtained from the genomes of strains MT-113^T^ and MT-5^T^. Genomic DNA extraction, PCR amplification and sequencing of the 16S rRNA gene followed previously established protocols [19]. Sequence similarities of the 16S rRNA gene were evaluated using the EzBioCloud server [25], with alignments performed via clustal W [26]. Phylogenetic trees were constructed using the neighbour-joining (NJ) method [27] with Kimura’s two-parameter model [28] in mega X [29], as well as the maximum-likelihood (ML) method [30] based on the Tamura-Nei model and the maximum-parsimony (MP) method [31] employing the Subtree-Pruning-Regrafting search method. The statistical robustness of the trees was assessed through bootstrap analysis with 1000 replications [32].

### Genome sequencing and analysis

Genomic DNA was extracted using the Wizard Genomic DNA Purification Kit (Promega) and subsequently sequenced on an Illumina Hiseq X-ten platform. High-quality paired reads were assembled using SPAdes software (v3.9.0) [33]. The completeness of each genome assembly was assessed with CheckM (version 1.1.6) [34]. The up-to-date bacterial core gene set UBCG (https://www.ezbiocloud.net/tools/ubcg) [35] was used to extract closely related genomes and construct a genome-based phylogenomic tree. The average nucleotide identity (ANI) among closely related genomes was determined using the OAT software (http://www.ezbiocloud.net/sw/oat), and a UPGMA dendrogram (unweighted pair group method with arithmetic mean) [36] was generated. The average amino acid identity (AAI) was calculated using EzAAI (http://leb.snu.ac.kr/ezaai) [37]. Genomic distances were measured as digital DNA–DNA hybridization (dDDH), using the Genome-To-Genome Distance Calculator (http://ggdc.dsmz.de/) [38]. Additionally, the percentage of conserved proteins was computed following an established method [39].

## Results

### Physiological and biochemical characteristics

Strains MT-113^T^ and MT-5^T^ were successfully cultured in mCGM medium [18]. Similar to *C. luticellarii* FW431^T^ [10] and * C. aromativorans* WLY-B-L2^T^ [11], MT-113^T^ and MT-5^T^ formed visible colonies on mCGM agar plates at 37 °C within 3 to 5 days. All strains exhibited ivory-coloured, round and convex colonies with smooth edges, ranging in diameter from 0.5 to 2.0 mm. Notably, MT-5^T^ exhibited larger colonies than MT-113^T^ within the same timeframe. Cell morphology was examined using TEM (see Fig. S1, available in the online Supplementary Material). Flagella were observed in the cells of MT-113^T^, while all strains displayed rod-shaped cells. MT-113^T^ cells measured 0.6 to 1.0 µm in width and 4.0 to 6.0 µm in length, which is slightly longer but thinner than MT-5^T^ (width: 1.0 to 1.2 µm, length: 3.0 to 4.0 µm). Growth analysis showed that MT-113^T^ thrived at temperatures between 20 and 45 °C (optimal at 37 °C), pH levels of 4.5 to 8.0 (optimal at pH 7.0) and up to 2.5% NaCl concentration (w/v; optimal at 0.5%). In contrast, MT-5^T^ grew at temperatures ranging from 20 to 45 °C (optimal at 37 °C), tolerated pH levels between 4.5 and 8.0 (optimal at pH 6.5) and up to 2% NaCl concentration (w/v; optimal at 0.5%) (see Table 1). While the optimal growth temperatures and NaCl tolerance were similar for all four strains, *C. luticellarii* FW 431^T^ and *C. aromativorans* WLY-B-L2^T^ had higher optimal pH values. None of the strains grew under aerobic conditions, indicating strict anaerobic requirements. Sensitivity testing showed that MT-113^T^ was susceptible to chloramphenicol, penicillin, polymyxin B, tetracycline and erythromycin, whereas MT-5^T^ was sensitive to ciprofloxacin, penicillin, amoxicillin, kanamycin and erythromycin.

**Table 1.** Phenotypic characteristics of strain MT-113^T^ and MT-5^T^ and closely related strains: Strains: 1. MT-113^T^; 2, MT-5^T^; 3, *C. aromativorans* WLY-B-L2^T^ [11]; 4, *C. luticellarii* FW431^T^ [10]

Characteristics	1	2	3	4
Isolation source	Pit mud	Pit mud	Pit mud	Mud cellar
Gram reaction	+	+	+	+
Cell morphology	Straight rods	Straight rods	Straight rods	Straight rods
Cell size (μm)	0.6–0.8*3.0–7.0	0.6–0.8*2.0–5.0	0.5–0.7*1.7–5.1	1–2*8–9
Temperature range (optimum) for growth (°C)	20–45 (37)	20–45 (37)	20–45 (37)	20–45 (37)
pH range (optimum) for growth	4.5–8.0 (7.0)	4.5–8.0 (6.5)	4.5–8.0 (5.5)	4.5–8.0 (5.0)
NaCl tolerance (%, w/v)	0–2.5	0–2	0–2.5	0–3
Substrates utilized:				
Dulcitol	−	−	−	+
d-Fructose	+	+	+w	+
l-Fucose	+w	+w	+w	+w
d-Galactose	+	+	−	−
d-Galacturonic acid	+	+	+	+
Gentiobiose	+	+	−	−
*α*-d-Glucose	+	+	−	−
Glycerol	+w	−	+	+
d,l-*α*-Glycerol phosphate	−	−	+w	+
Lactulose	+w	+w	−	−
d-Mannose	+	+	−	−
d-Melibiose	+w	+	−	−
3-Methyl-d-glucose	+	+	−	+
*α*-Methyl-d-galactoside	−	−	+w	+
Palatinose	+	+	+w	+w
l-Rhamnose	+w	+w	−	−
d-Sorbitol	−	−	+w	+
Turanose	+w	+	+w	+
Glyoxylic acid	+w	+w	−	−
*β*-Hydroxybutyric acid	+	+w	−	+
d-Lactic acid methyl ester	−	−	+w	+
d-Malic acid	−	−	−	+
l-Malic acid	−	−	−	+
Pyruvic acid	+w	+w	+	+
Pyruvic acid methyl ester	+w	+w	+	+
l-Alanine	+	+w	+	+
l-Alanyl-l-glutamine	−	−	+	+w
l-Alanyl-l-histidine	+w	−	+	−
l-Alanyl-l-threonine	−	−	+	−
l-Methionine	−	+w	+	+
l-Phenylalanine	+w	+w	+	+
l-Serine	−	−	+	+
l-Threonine	−	−	−	+
l-Valine	−	−	+	−
l- Valine plus l-aspartic acid	−	+w	+	+w
Thymidine	−	−	+	+w
Fermentation end-products	Acetic acid, butyric acid, isovaleric acid	Acetic acid, isobutyric acid, butyric acid, isovaleric acid	Acetic acid, isobutyric acid, butyric acid, isovaleric acid, valeric acid, hexanoic acid	Acetic acid, butyric acid, isovaleric acid
Major fatty acids (>10%)	C14 : 0, C16 : 0, Summed feature 1	C14 : 0, Summed feature 1, Summed feature 3	C14 : 0, C16 : 0, Summed feature 3	C14 : 0, C16 : 0
Data from genome analysis:*				
Genome size (Mb)	3.21	3.66	3.88	3.75
DNA G+C (mol%)	35.84	32.74	34.2	34.97

For strains 3 and 4, most data were obtained in our study (except *, obtained from the genome data in the database [10,11]). +, positive; w+, weakly positive; −, negative.

In the Biolog AN MicroPlate assay, strains MT-113^T^ and MT-5^T^ assimilated 11 common carbon sources, including d-fructose, d-galactose, d-galacturonic acid, gentiobiose, *α*-d-glucose, d-mannose, 3-methyl-d-glucose, palatinose, turanose, *β*-hydroxybutyric acid and l-alanine. Mono- and disaccharides were preferred substrates, which are abundant in pit mud environments. Results from the Rapid ID 32A test indicated that MT-113^T^ exhibited positive traits for mannose and raffinose fermentation, glutamic acid decarboxylase and proline arylamidase, while MT-5^T^ showed similar traits except for proline arylamidase. The API ZYM test demonstrated that MT-113^T^ and MT-5^T^ shared enzymatic reactions with *C. aromativorans* WLY-B-L2^T^ and *C. luticellarii* FW431^T^, particularly in esterase (C4), esterase-lipase (C8), acid phosphatase and naphthol-AS-Bi-phosphohydrolase substrates. Analysis of short-chain fatty acid production in glucose-supplemented RCM broth revealed the production of acetic, butyric and isovaleric acids by MT-113^T^, MT-5^T^, *C. aromativorans* WLY-B-L2^T^ and *C. luticellarii* FW431^T^. Additionally, MT-5^T^ produced isobutyric acid, while *C. aromativorans* WLY-B-L2^T^ also synthesized isobutyric, valeric and hexanoic acids. Distinctive characteristics between MT-113^T^, MT-5^T^ and closely related strains are detailed in Table 1, while comprehensive physiological traits of MT-113^T^ and MT-5^T^ are provided in the species description.

### Cellular fatty acids and polar lipid profiling

Chemotaxonomic analysis of cellular fatty acids and polar lipid profiles for strains MT-113^T^ and MT-5^T^ are presented in Tables 1 and S2. Strain MT-113^T^ predominantly exhibited fatty acids (>10%) such as C_14 : 0_ (51.0%), C_16 : 0_ (13.3%) and summed feature 1 (iso-C_15 : 1_ h and/or C_13 : 0_ 3-OH, 19.6%), which together constituted 83.9% of the total fatty acids. By contrast, the major fatty acids (>10%) in strain MT-5^T^ included C_14 : 0_ (43.9%), summed feature 1 (iso-C_15 : 1_ h and/or C_13 : 0_ 3-OH, 18.5%) and summed feature 3 (C_16 : 1_ ω6c and/or C_16 : 1_ ω7c, 13.6%), comprising 73.6% of the total fatty acids. The distinct production of C_16 : 0_ and summed features 1 and 3 facilitated differentiation among MT-113^T^, MT-5^T^, *C. aromativorans* WLY-B-L2^T^ and *C. luticellarii* FW431^T^. Furthermore, MT-113^T^ exhibited C_15 : 1_ ω5c and summed feature 4 (iso-C_17 : 0_ I and/or anteiso-C_17 : 1_ B), which were absent in the other three strains, whereas only *C. aromativorans* WLY-B-L2^T^ contained cyclo-C_17 : 0_. The predominant polar lipids in MT-113^T^, MT-5^T^, *C. aromativorans* WLY-B-L2^T^ and *C. luticellarii* FW431^T^ were diphosphatidylglycerol (DPG), phosphatidylglycerol (PG) and phosphatidylethanolamine (PE). Variations were observed in the presence of unidentified PLs and unidentified GLs, as detailed in Fig. S2.

### Phylogeny and genome features

**Fig. 1.**
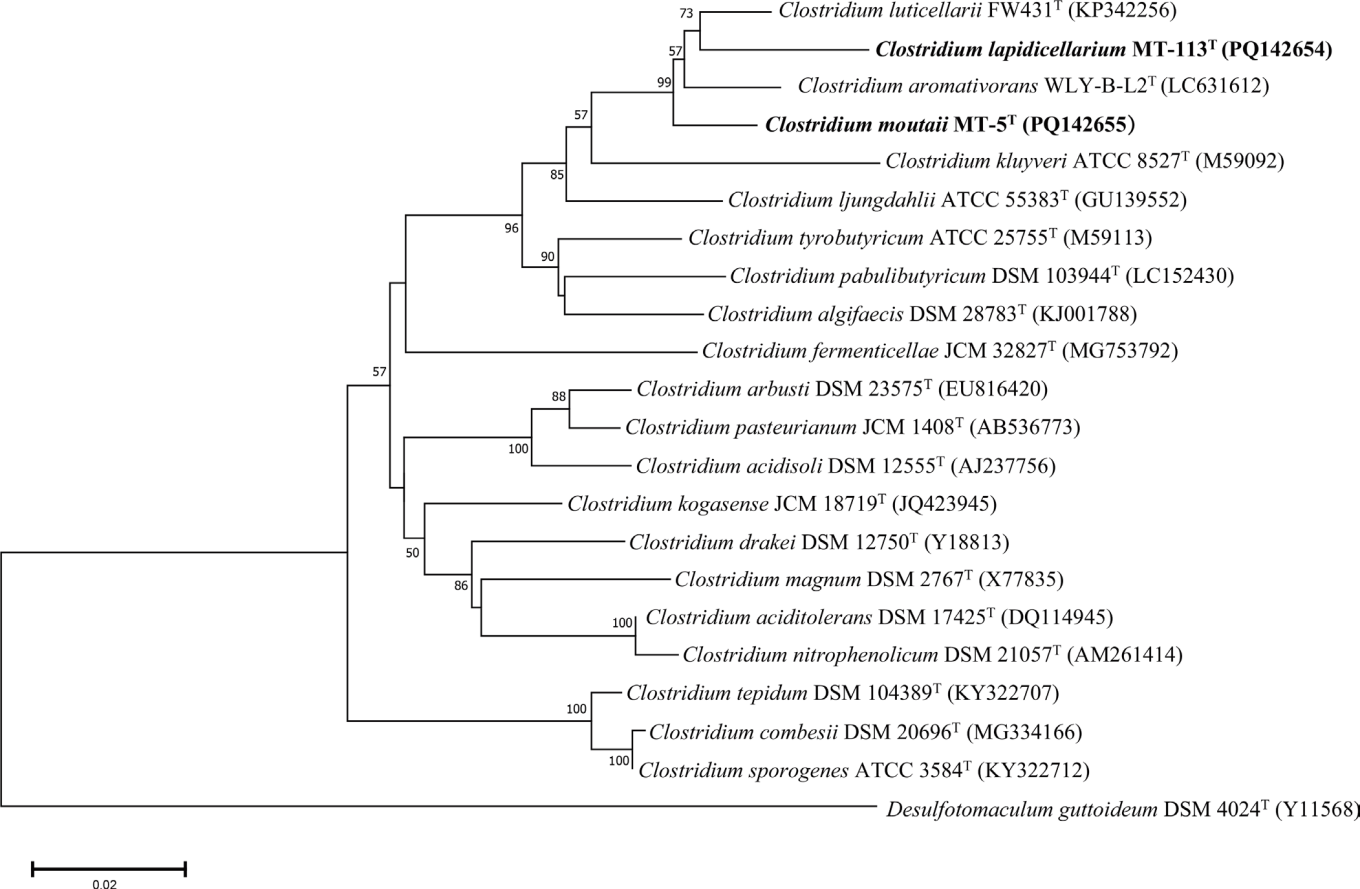
NJ phylogenetic tree based on 16S rRNA gene sequences showing the position of strains MT-113^T^ and MT-5^T^ among the species of the genus *Clostridium*. Bootstrap values that were above 50% based on 1000 replicates bootstrap samplings were shown at the branch points. *Desulfotomaculum guttoideum* DSM 4024^T^ (Y11568) was used as outgroup. Bar represents 0.02 substitutions per nucleotide position.

**Fig. 2.**
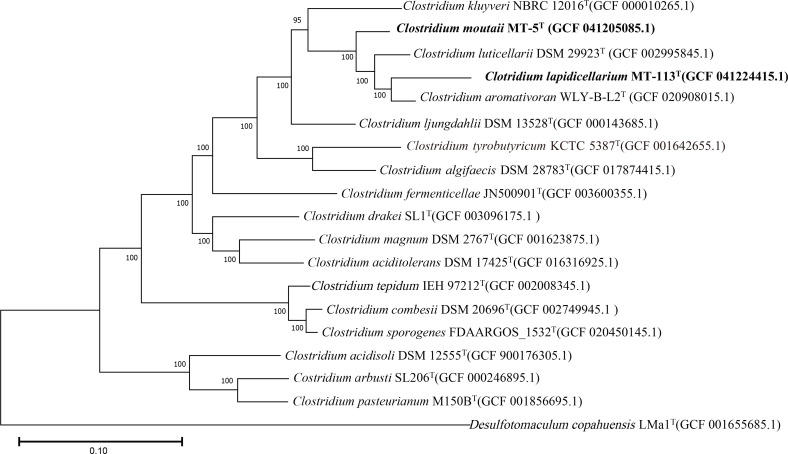
Phylogenetic relationships at the genomic level of strains MT-113^T^ and MT-5^T^ and the species of the genus *Clostridium*. Phylogenomic tree of each strain and its closely related strains based on 92 bacterial core gene sequences constructed using UBCGs. GenBank accession numbers of the genomes used are given in parentheses. The gene support indices indicating the number of single gene trees supporting each branch in the tree from the concatenated alignment are marked on the branches. *Desulfotomaculum copahuensis* LMa1^T^ (GCF 001655685.1) was used as outgroup. Bars, 0.10 substitutions per site.

The full-length 16S rRNA gene sequences of strains MT-113^T^ (1507 bp) and MT-5^T^ (1508 bp) have been submitted to GenBank under accession numbers PQ142654 and PQ142655, respectively. Pairwise comparisons using the EzBioCloud database identified *C. luticellarii* FW431^T^ (96.58%), *C. aromativorans* WLY-B-L2^T^ (96.23%), MT-5^T^ (95.74%) and *C. ljungdahlii* DSM13528^T^ (94.13%) as the closest phylogenetic relatives to strain MT-113^T^. Similarly, MT-5^T^ showed close relationships with *C. aromativorans* WLY-B-L2^T^ (97.42%), *C. luticellarii* FW431^T^ (97.35%), *C. ljungdahlii* DSM13528^T^ (96.37%) and MT-113^T^ (95.74%). The 16S rRNA gene phylogenetic tree, constructed using the NJ method, demonstrated that MT-113^T^ and MT-5^T^ formed a clade with *C. luticellarii* FW431^T^ and *C. aromativorans* WLY-B-L2^T^ (Fig. 1). This tree’s stability was corroborated by the topologies of the ML and MP trees (Figs S3 and S4). Furthermore, a phylogenomic tree based on the alignment of 92 marker genes provided additional evidence supporting the distinct lineages of MT-113^T^ and MT-5^T^ (Fig. 2). Phylogenetic and phylogenomic analyses indicated that strains MT-113^T^ and MT-5^T^ represent novel species within the genus *Clostridium*.

**Fig. 3.**
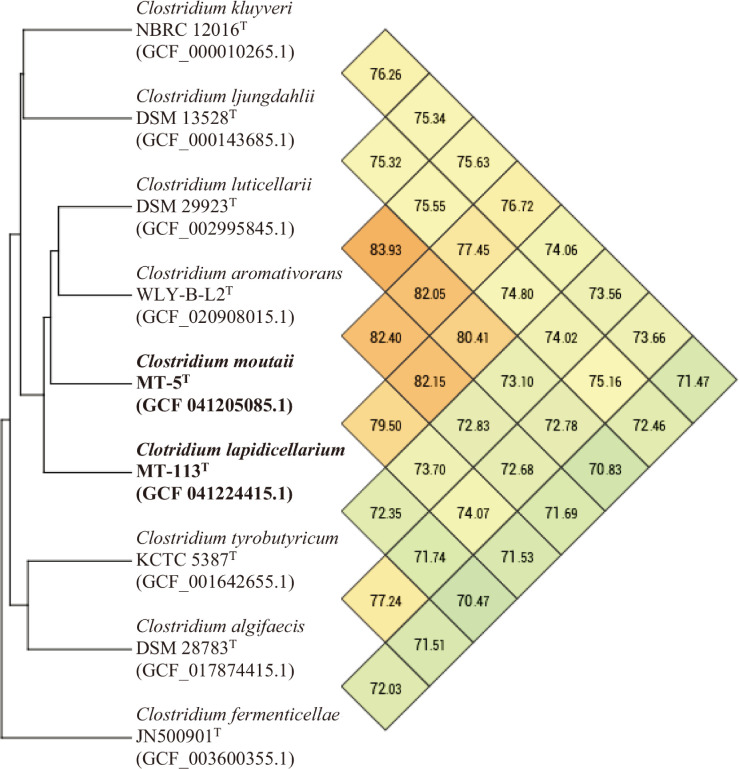
UPGMA phylogenetic trees and ANI heat maps based on whole genomes. UPGMA phylogenetic trees and the ANI heat maps display the connections between MT-113^T^ and MT-5^T^ and their respective closely related neighbours, respectively. GenBank accession numbers of the genomes are shown in parentheses.

Analysis of ANI and AAI identified *C. aromativorans* WLY-B-L2^T^, *C. luticellarii* FW431^T^ and MT-5^T^ as the closest species to strain MT-113^T^ (Figs 3 and S5). The dDDH values for MT-113^T^ with *C. aromativorans* WLY-B-L2^T^ and *C. luticellarii* FW431^T^ were 25.20 and 23.40%, respectively. For MT-5^T^, the ANI and AAI analyses identified *C. aromativorans* WLY-B-L2^T^ as the closest relative, followed by *C. luticellarii* FW431^T^ and MT-113^T^ (Figs 3 and S5). The dDDH values for MT-5^T^ with *C. aromativorans* WLY-B-L2^T^ and *C. luticellarii* FW431^T^ were 25.40 and 25.10%, respectively. As the ANI/AAI and dDDH values for both MT-113^T^ and MT-5^T^ fall below the 95.0 and 70.0% thresholds, respectively, for species identification, we propose that these strains represent novel species within the genus *Clostridium*.

The draft genome of strain MT-113^T^ comprised 111 contigs in 81 scaffolds, totalling 3 213 286 bp with an N50 length of 103 000 bp. It contained 3103 predicted genes, inclusive of 61 tRNA genes and 3 rRNA genes. Similarly, the MT-5^T^ draft genome, comprising 81 contigs across 56 scaffolds and totalling 3 586 104 bp with an N50 length of 203 880 bp, harbours 3507 predicted genes, which include 65 tRNA genes and 4 rRNA genes. The G+C content of MT-113^T^ and MT-5^T^ is 35.84 and 32.74%, respectively, aligning with the typical G+C content range of 21–54 mol% in the *Clostridium* genus [40]. The Kyoto Encyclopedia of Genes and Genomes (KEGG) pathway analysis presented in Table S1 indicates that both strains predominantly feature genes for carbohydrate metabolism, followed by amino acid, cofactor and vitamin and energy metabolism, corresponding to the gene profiles of *C. aromativorans* WLY-B-L2^T^ and *C. luticellarii* FW431^T^.

The study compared the carbon source utilization characteristics, major polar lipid compositions and cellular fatty acid proportions of strains MT-113^T^ and MT-5^T^, detailed in Tables 1 and S2 and Figs S2 and S5. Analyses of the 16S rRNA gene and genome sequences revealed low similarities to known phylogenetic neighbours, suggesting that these strains constitute two novel species within the *Clostridium* genus, designated as *Clostridium lapidicellarium* sp. nov. and *Clostridium moutaii* sp. nov., respectively.

## DESCRIPTION OF *CLOSTRIDIUM LAPIDICELLARIUM* SP. NOV.

*Clostridium lapidicellarium* sp. nov. (la.pi.di.cel.la’ri.um. L. masc. n. *lapis*, stone; L. masc. adj. *cellarius*, relating to a storeroom; N.L. neut. adj. *lapidicellarium*, of a stone cellar).

Cells are Gram-positive, straight rods measuring 0.6–0.8 µm in width and 3–7 µm in length, equipped with flagella. After 3–5 days of incubation at 37 °C on mCGM agar plates, it forms circular, convex, ivory-coloured colonies with smooth, entire edges, measuring 0.5–1.0 mm in diameter. Growth occurs at temperatures ranging from 20 to 45 °C and at pH levels between 4.5 and 8, with optimal growth at 37 °C and pH 7. The primary fermentation products from glucose are acetic, butyric and isovaleric acids when cultured on RCM. Optimal growth is observed at 0.5% (w/v) NaCl, with no growth observed at NaCl concentrations of 2.5% (w/v) or higher. The Biolog test confirms the utilization of d-fructose, d-galactose, gentiobiose, d-galacturonic acid, *α*-d-glucose, d-mannose, 3-methyl-d-glucose, palatinose, *β*-hydroxybutyric acid and l-alanine as sole carbon sources. The organism shows positive Rapid ID 32A and API ZYM reactions for the fermentation of mannose and raffinose, as well as the activities of glutamic acid decarboxylase, proline arylamidase, esterase (C4), esterase-lipase (C8), acid phosphatase and naphthol-AS-BI-phosphohydrolase. It is sensitive to the antibiotics chloramphenicol, penicillin, polymyxin B, tetracycline and erythromycin. The major cellular fatty acids are C_14 : 0_, C_16 : 0_ and summed feature 1. The polar lipid profile comprises DPG, PG and PE, along with three unidentified PLs and two unidentified GLs. The genome size is 3 213 286 bp, with a G+C content of 35.84 mol%. The GenBank accession number for the 16S rRNA and whole genome sequences of strain MT-113^T^ are PQ142654 and JBGFFE000000000, respectively.

The name *Clostridium lapidicellarium* sp. nov. is proposed, with the type strain designated as MT113^T^ (=CGMCC 1.18018^T^ = JCM 36532^T^), which was isolated from pit mud in a stone cellar used for the production of sauce-flavour Chinese baijiu.

## DESCRIPTION OF *CLOSTRIDIUM MOUTAII* SP. NOV.

*Clostridium moutaii* sp. nov. (mou’tai.i. N. L. neut. n. *moutaii*, referring to Moutai, a prominent liquor manufacturer in China, from whose production site the type strain was isolated).

Cells are Gram-positive, strictly anaerobic, non-motile bacterium characterized by straight rod-shaped cells ~0.6–0.8 µm wide and 2.0–5.0 µm long. Strain MT-5^T^ forms round, convex, ivory-coloured colonies measuring 1–3 mm in diameter after 3 days of incubation at 37 °C on mCGM agar plates. Growth occurs between temperatures of 20 and 45 °C, with optimal growth at 37 °C; the pH range is 4.5–8, with optimal growth at a pH of 6.5; and tolerable NaCl concentrations are 0–2% (w/v). The primary fermentation products from glucose are acetic, isobutyric, butyric and isovaleric acids when cultured on RCM. The Biolog test indicates positive results for the utilization of d-fructose, d-galactose, gentiobiose, d-galacturonic acid, α-d-glucose, d-mannose, d-melibiose, 3-methyl-d-glucose, palatinose and turanose as sole carbon sources. The Rapid ID 32A and API ZYM tests show positive reactions for the fermentation of mannose and raffinose and for the activity of glutamic acid decarboxylase, esterase (C4), esterase-lipase (C8), acid phosphatase and naphthol-AS-BI-phosphohydrolase. The bacterium is sensitive to ciprofloxacin, penicillin, amoxicillin, kanamycin and erythromycin. Major cellular fatty acids include C_14 : 0_, summed feature 1 and summed feature 3. The polar lipids repertoire comprises DPG, PG and PE, while the minor lipids consist of three unidentified PLs and three unidentified GLs. The genome size is 3 661 612 bp, and the G+C content is 32.74 mol%. The GenBank accession number for the 16S rRNA and whole-genome sequences of strain MT-5^T^ are PQ142655 and JBGEWD000000000, respectively.

The name *Clostridium moutaii* sp. nov. is proposed, with the type strain designated as MT-5^T^ (=CGMCC 1.18016^T^ = JCM 36530^T^), which was isolated from pit mud used for the production of sauce-flavour Chinese baijiu.

## supplementary material

10.1099/ijsem.0.006580 Uncited Supplementary Material 1.

## Abbreviations

ANI: average nucleotide identity
dDDH: DNA–DNA hybridization
ML: maximum-likelihood
MP: maximum-parsimony
NJ: neighbour-joining
TEM: transmission electron microscopy
