# Supplementary material for: Clostridium lapidicellarium sp. nov. and Clostridium moutaii sp. nov., two species isolated from fermentation cellar-producing sauce-flavour Chinese baijiu
Source: Int J Syst Evol Microbiol. 2024 Nov 19;74(11):006580. doi: 10.1099/ijsem.0.006580 (PMC11648564; doi:10.1099/ijsem.0.006580)
Supplement: Uncited Supplementary Material 1. [file ijsem-74-06580-s001.pdf]

Clostridium lapidicellarium sp. nov., and *Clostridium moutaii* sp. nov., two species isolated from the mud in a fermentation cellar used for producing sauce-flavor Chinese baijiu.

Fang Yang[1], Hui Wang[1], Liang-Qiang Chen[1], Nan Zhou[2], Jian-Jun Lu[1], Xiu-Xin Pu[1], Bo Wang[1], Li Wang[1]*, Shuang-Jiang Liu[2,3]*

[1]Kweichow Moutai Distillery Co., Ltd., Zunyi 564501, China.

[2]State Key Laboratory of Microbial Resources, and Environmental Microbiology Research Center (EMRC), Institute of Microbiology, Chinese Academy of Sciences, Beijing, 100101, P. R. China

[3]State Key Laboratory of Microbial Technology, Shandong University, Qingdao 266237, P. R. China.

*Authors for correspondence: Shuang-Jiang Liu (liusj@im.ac.cn or liusj@sdu.edu.cn) and Li Wang (wanglimoutai2021@163.com); Postal address: Institute of Microbiology, Chinese Academy of Sciences, Beichen West Road No. 1, Chaoyang District, Beijing 100101, China;

Running title: *Clostridium lapidicellarium* sp. nov., *and Clostridium renhuaiense* sp. nov.,

Keywords: *Clostridium sensu stricto* (cluster I); *C. lapidicellarium*; *C. moutaii*; pit mud; sauce-flavor Chinese *baijiu*

Contents category: New Taxa; Subsection: *Clostridium*

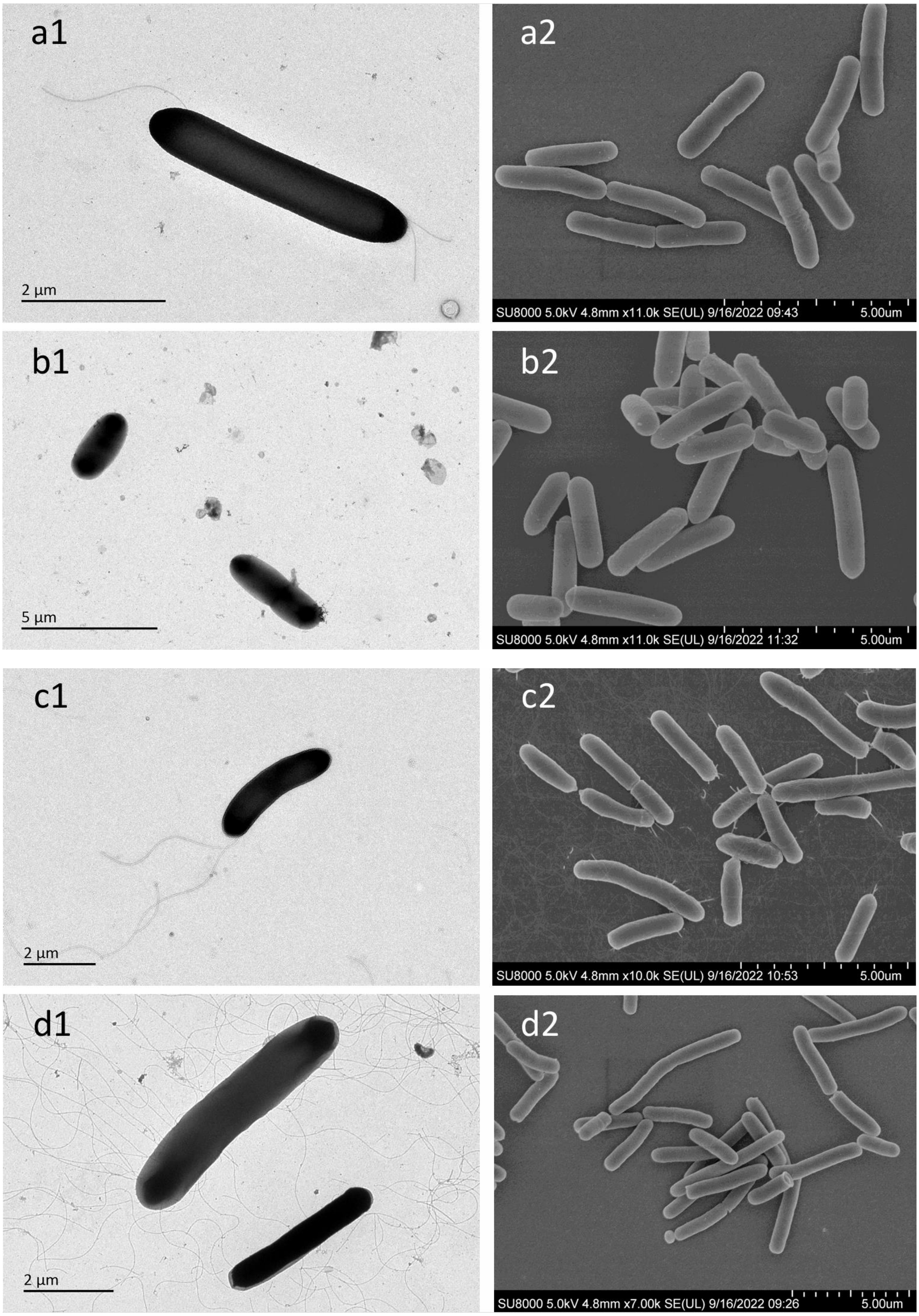

Fig. S1. Micrographs of MT-113^T cells (a1, a2), MT-5^T cells (b1, b2), *C.aromativorans* WLY-B-L2^T(c1,c2) and *C.luticellarii* FW431^T (d1,d2). Transmission electron microscope (TEM) showing the flagella (a1, c1, d1).

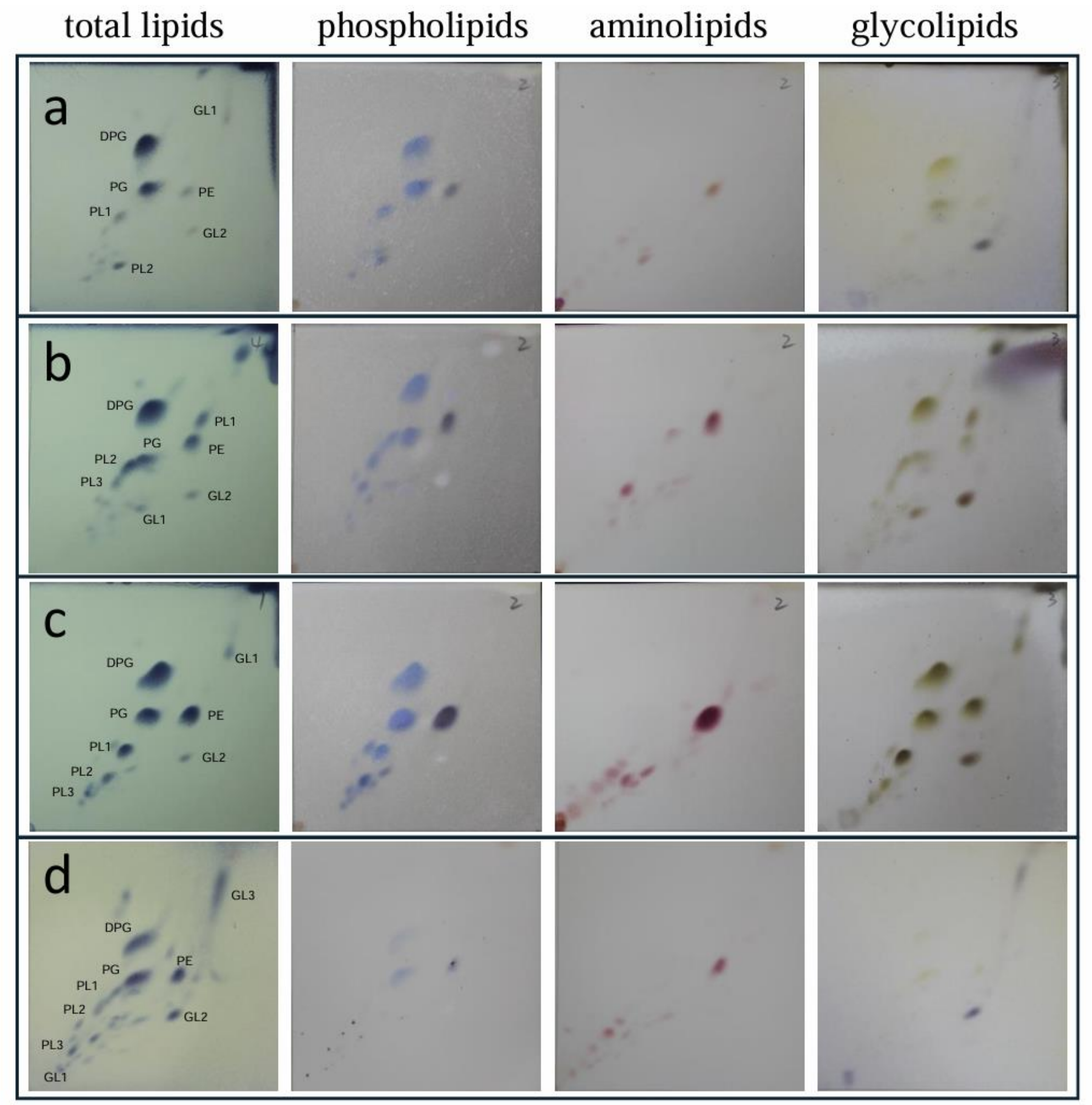

Fig. S2. Polar lipids profile of strain MT-113^T (a), MT-5^T (b), *C.aromativorans* WLY-B-L2^T(c) and *C.luticellarii* FW431^T (d), following two-dimensional TLC and stained with molybdatophosphoric acid.

Abbreviations: DPG, diphosphatidylglycerol; PE, phosphatidylethanolamine; PG, phosphatidylglycerol; GL, unidentified glycolipid; and PL, unidentified phospholipid.

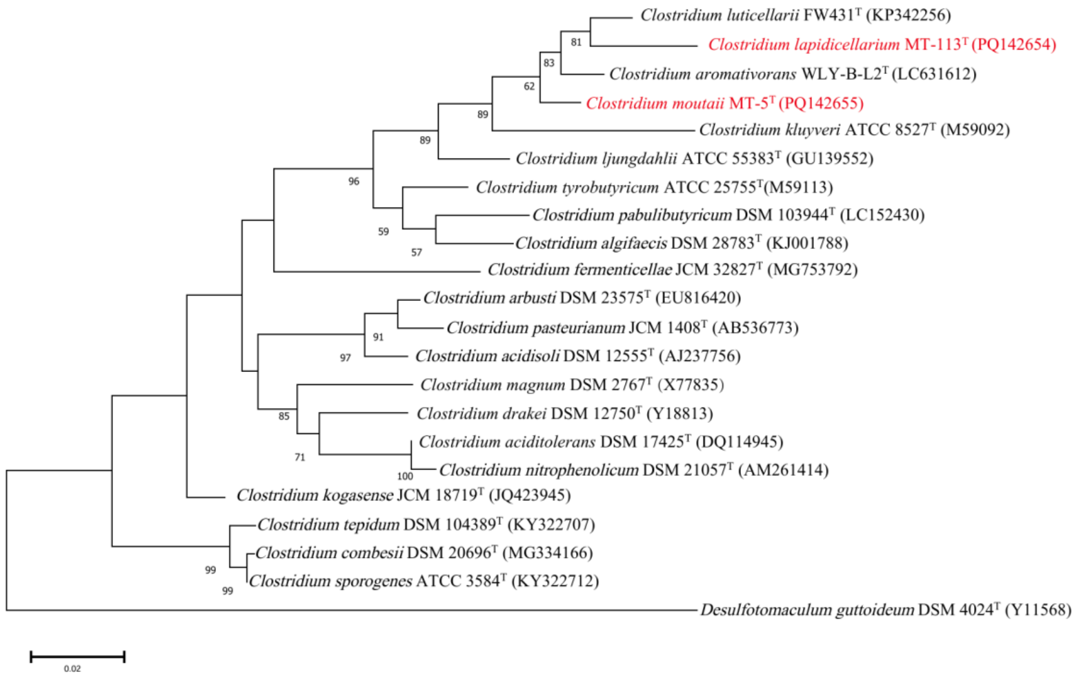

Fig. S3. Maximum-likelihood phylogenetic tree based on 16S rRNA gene sequences showing the position of strains MT-113^{T} and MT-5^{T} among the genus *Clostridium*. Sequences were downloaded from NCBI. Bootstrap values of above 50% are shown at the branch points. *Desulfotomaculum guttoideum* DSM 4024^{T}(Y11568) was used as outgroup. Bar represents 0.02 substitutions per nucleotide position.

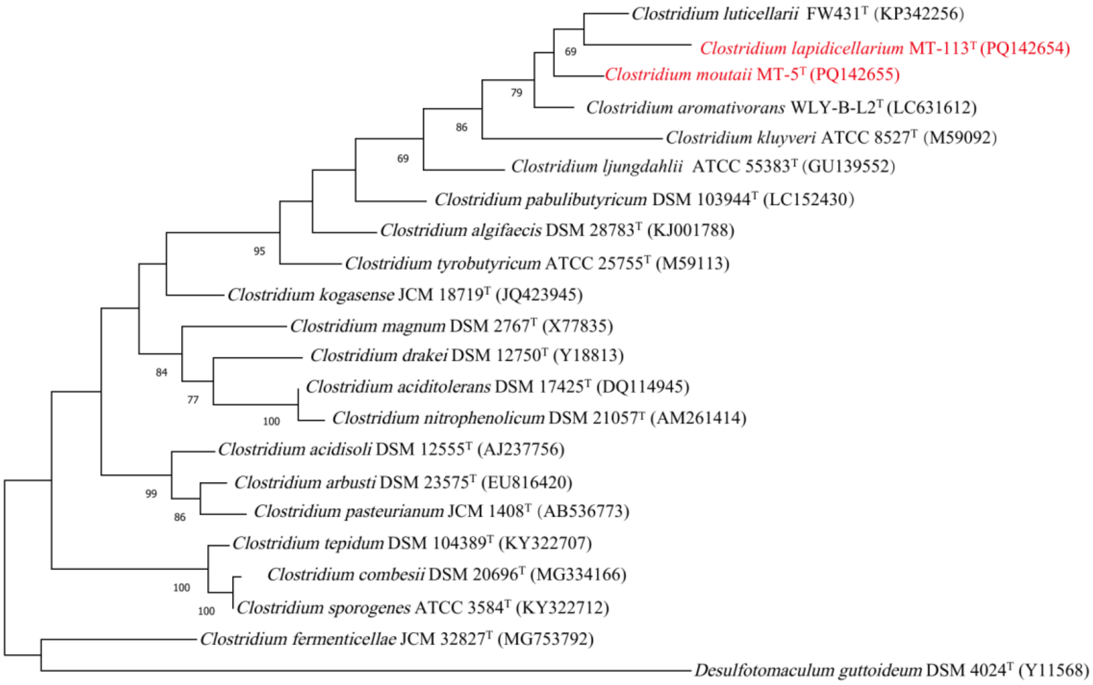

Fig. S4. Maximum-parsimony phylogenetic tree based on 16S rRNA gene sequences showing the position of strains MT-113^{T} and MT-5^{T} among the genus Clostridium. Sequences were downloaded from NCBI. Bootstrap values of above 50 % are shown at the branch points. *Desulfotomaculum guttoideum* DSM 4024T(Y11568) was used as the outgroup.

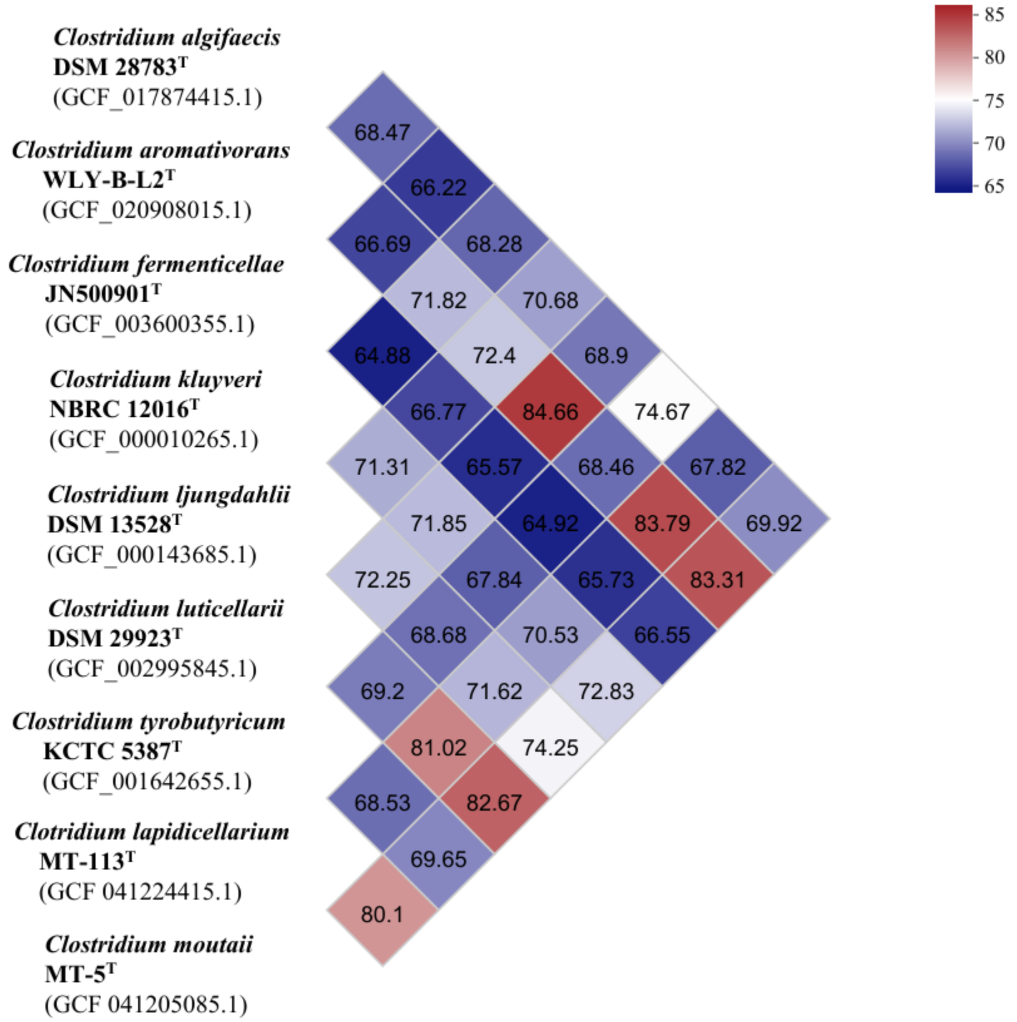

Fig. S5. AAI heat maps based on whole genomes. AAI heat maps display MT-113^{T} and MT-5^{T} and their respective closely related neighbours, respectively. GenBank accession numbers of the genomes are shown in parentheses.

Table S1. Number of genes associated with the KEGG functional pathways.

Strains: 1, MT-113^T; 2, MT-5^T; 3, *C.aromativorans* WLY-B-L2^T[11]; 4, *C.luticellarii* FW431^T[10]

KEGG functional pathways	1	2	3	4
Cell motility	47	55	47	55
Cell growth and death	20	21	19	17
Cellular community - prokaryotes	48	60	52	60
Transport and catabolism	4	4	4	3
Biosynthesis of other secondary metabolites	37	33	35	34
Xenobiotics biodegradation and metabolism	33	48	49	54
Lipid metabolism	47	56	49	49
Carbohydrate metabolism	171	178	212	192
Metabolism of cofactors and vitamins	125	142	136	141
Amino acid metabolism	169	183	196	174
Glycan biosynthesis and metabolism	45	43	49	51
Nucleotide metabolism	66	77	72	68
Energy metabolism	127	149	159	171
Metabolism of other amino acids	33	33	34	30
Metabolism of terpenoids and polyketides	20	18	25	24
Translation	80	80	81	78
Replication and repair	48	53	68	48
Folding, sorting and degradation	47	50	50	47
Transcription	4	4	4	4
Signal transduction	90	93	94	90
Membrane transport	84	87	93	103
Global and overview maps	581	620	653	632

Table S2. Fatty acid compositions of strains MT-113^T and MT-5^T and their closely related Strains: Strain 1, MT-113^T; 2, MT-5^T; 3, *C.aromativorans* WLY-B-L2^T; 4, *C.luticellarii* FW431^T. All data were obtained in this study. Only fatty acids that accounted for >0.5 % in at least one of the strains are listed. –, not detected. Fatty acids present at >10.0 % are highlighted in bold.

Fatty acid	1	2	3	4
$C_{12:0}$	1.1	0.4	0.9	—
$C_{13:1}$	—	7.0	3.1	2.4
$C_{14:0}$	**51.0**	**43.9**	**40.8**	**18.5**
iso-$C_{15:0}$	0.5	0.1	—	—
$C_{15:1}\omega5c$	2.1	—	—	—
$C_{15:1}\omega8c$	—	2.7	0.5	1.2
$C_{16:0}$	**13.3**	8.4	**15.5**	**60.5**
iso-$C_{17:1}\omega5c$	—	—	1.9	—
cyclo-$C_{17:0}$	—	—	6.2	—
$C_{17:1}\omega9c$	0.2	0.5	—	—
$C_{17:0}$	—	—	0.7	—
$C_{18:1}\omega9c$	—	0.4	3.4	1.8
$C_{18:0}$	—	0.3	1.8	2.2
Summed Feature*				
1	**19.6**	**18.5**	8.6	7.3
3	4.6	**13.6**	**12.1**	4.1
4	2.4	—	—	—
8	—	0.5	2.5	2.0

* Summed Features are fatty acids that cannot be resolved reliably from another fatty acid using the chromatographic conditions chosen. The MIDI system groups these fatty acids together as one feature with a single percentage of the total. Summed feature 1 comprises iso-$C_{15:1}$ H and/or $C_{13:0}$ 3-OH; summed feature 3 comprises $C_{16:1}\omega6c$ and/or $C_{16:1}\omega7c$; summed feature 4 comprises iso-$C_{17:0}$ I and/or anteiso-$C_{17:1}$ B; summed feature 8 comprises $C_{18:1}\omega7c$ and/or $C_{18:1}\omega6c$.
